# Supplementary material for: CRISPR/Cas9-engineered inducible gametocyte producer lines as a valuable tool for Plasmodium falciparum malaria transmission research
Source: Nat Commun. 2021 Aug 10;12:4806. doi: 10.1038/s41467-021-24954-4 (PMC8355313; doi:10.1038/s41467-021-24954-4)
Supplement: Supplementary file 1 — Supplementary Information [file 41467_2021_24954_MOESM1_ESM.pdf]

Supplementary Information

CRISPR/Cas9-engineered inducible gametocyte producer lines as a novel tool for basic and applied research on *Plasmodium falciparum* malaria transmission stages

Sylwia D. Boltryk[1,2], Armin Passecker[1,2], Arne Alder[3,4,5], Eilidh Carrington[1,2], Marga van de Vegte-Bolmer[6], Geert-Jan van Gemert[6], Alex van der Starre[6], Hans-Peter Beck[1,2], Robert W. Sauerwein[6], Taco W. A. Kooij[6], Nicolas M. B. Brancucci[1,2], Nicholas I. Proellochs[6], Tim-Wolf Gilberger[3,4,5], Till S. Voss[1,2,*]

[1]Department of Medical Parasitology and Infection Biology, Swiss Tropical and Public Health Institute, 4051 Basel, Switzerland.

[2]University of Basel, 4001 Basel, Switzerland.

[3]Centre for Structural Systems Biology, 22607 Hamburg, Germany.

[4]Bernhard Nocht Institute for Tropical Medicine, 20359 Hamburg, Germany.

[5]University of Hamburg, 20146 Hamburg, Germany.

[6]Department of Medical Microbiology, Radboudumc Center for Infectious Diseases, Radboud Institute for Molecular Life Sciences, Radboud University Medical Center, 6525 GA Nijmegen, The Netherlands.

[*]Corresponding author: till.voss@swisstph.ch.

This file includes:

- Supplementary Note 1
- Supplementary Figures 1 to 10
- Supplementary Tables 1 and 2

Supplementary Note 1. CRISPR/Cas9-based engineering of the 3D7/iGP inducible gametocyte producer line.

After co-transfection of the pHF_gC-*cg6* CRISPR/Cas9 plasmid and the pD_*cg6_cam-gdv1-gfp-dd* donor plasmid into 3D7 wild type parasites (Supplementary Fig. 1a), transgenic 3D7/iGP parasites were successfully selected on WR99210. PCRs on genomic DNA (gDNA) confirmed complete disruption of the *cg6* locus through insertion of the *gdv1-gfp-dd* donor assembly (Supplementary Fig. 1b). At this stage, both transfected plasmids were still detectable in the population and the majority of parasites carried integrated donor plasmid concatamers (Supplementary Figs. 1b-d), which has also been reported in other studies [1,2]. Treatment with 5-fluorocytosine (5-FC) successfully depleted parasites expressing the hDHFR-yFCU marker and enriched for parasites carrying a single GDV1-GFP-DD expression cassette integrated into the *cg6* locus (Supplementary Figs. 1b-d). This population was then used to obtain three 3D7/iGP clonal lines by limiting dilution cloning. PCRs on gDNA demonstrated that clones 3D7/iGP_B9 and 3D7/iGP_F10 were plasmid- and marker-free parasites carrying a single integrated GDV1-GFP-DD expression cassette (Supplementary Figs. 1c and 1d). Clone 3D7/iGP_D9 was also marker-free but still contained a donor plasmid concatamer in the *cg6* locus (Supplementary Figs. 1c and 1d).

To test if Shield-1 induces GDV1-GFP-DD overexpression and sexual conversion, we split ring stage parasites of the 5-FC-treated 3D7/iGP line at 8-16 hours post-invasion (hpi) (generation 1) into two equal populations and added Shield-1 to one of them (Fig. 1b). Indirect immunofluorescence assays (IFA) on schizont stage parasites (40-48 hpi, generation 1) showed that most parasites in the Shield-1-treated population expressed GDV1-GFP-DD, in contrast to parasites cultured in the absence of Shield-1 where GDV1-GFP-DD expression was hardly detectable (Supplementary Fig. 2a). IFAs probing for expression of the gametocyte marker Pfs16 [3] in the early stage I gametocyte progeny (36-44 hpi, generation 2; day 2 of gametocytogenesis) revealed mean sexual conversion rates (SCRs) of 69.0% (±4.5 s.d.), 75.2% (±1.4 s.d.) and 67.5% (±0.7 s.d.) for parasites cultured in the presence of 1350 nM, 675 nM and 337.5 nM Shield-1, respectively, compared to only 5.2% (±1.9 s.d.) for control parasites cultured in the absence of Shield-1 (Figs. 1b-d). Furthermore, all three 3D7/iGP clones showed high SCRs comparable to those achieved with the 3D7/iGP mother line (Supplementary Fig. 2b). To test if Shield-1-induced 3D7/iGP early stage gametocytes complete sexual differentiation, the sexual ring stage progeny were exposed to 50 mM N-acetylglucosamine (GlcNAc) for six consecutive days (days 1-6 of gametocytogenesis) to eliminate asexual parasites [4,5] and were thereafter maintained under routine culture conditions. Inspection of Giemsa-stained blood smears prepared on days 6 and 10 of gametocyte maturation revealed pure and synchronous stage III and V gametocyte populations, respectively (Supplementary Fig. 2c). Hence, by integrating a conditional GDV1-GFP-DD overexpression cassette into the 3D7 genome we obtained a stable 3D7/iGP line and marker-free clones suitable for the production of synchronous gametocyte cultures at high yield.

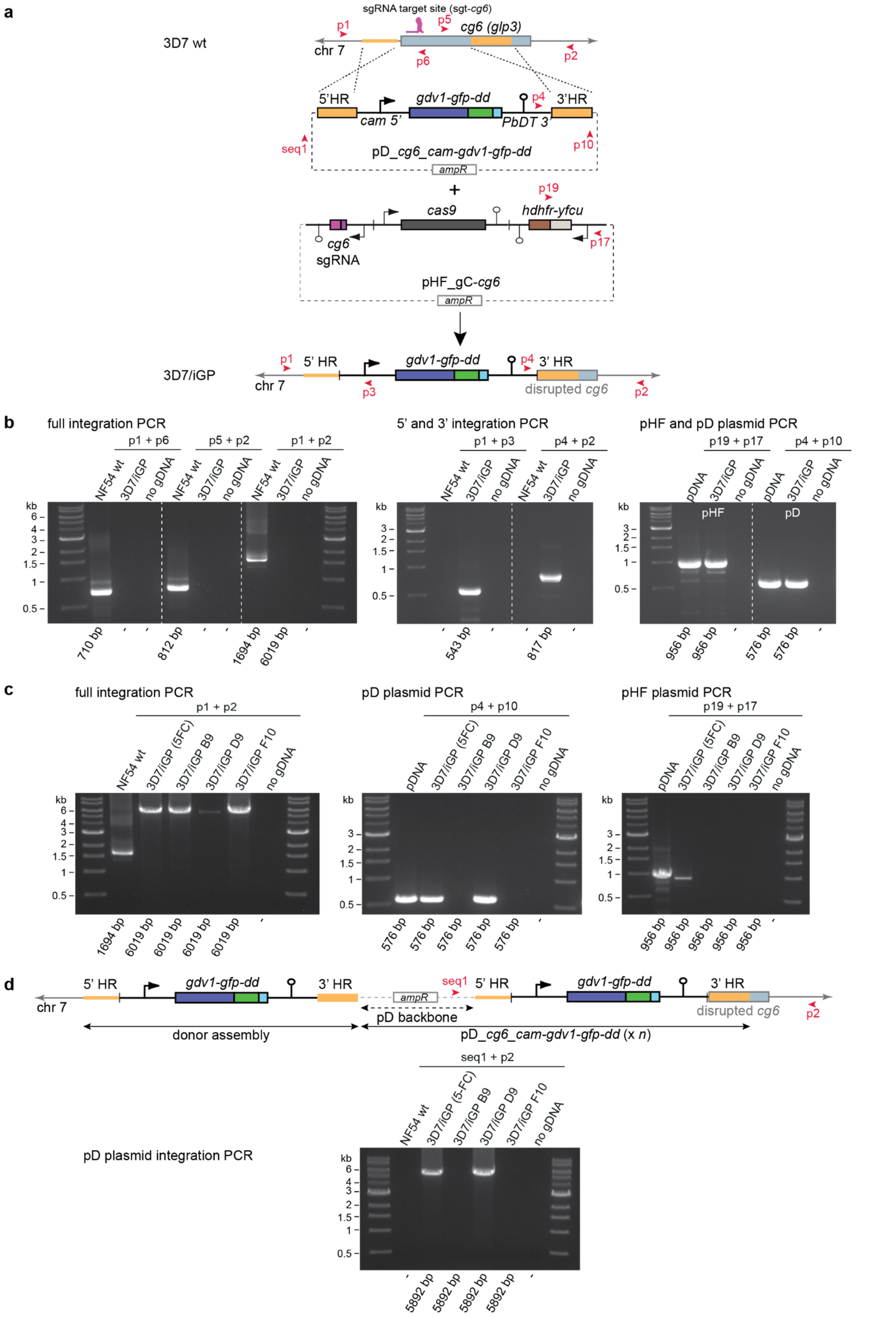

Supplementary Figure 1. CRISPR/Cas9-based engineering of the 3D7/iGP line. a Schematic maps of the endogenous *cg6* (*glp3*) locus (PF3D7_0709200) in 3D7 wild type (wt) parasites (top), the

pD_*cg6_cam-gdv1-gfp-dd* donor and pHF_gC-*cg6* CRISPR/Cas9 transfection plasmids (center), and the disrupted *cg6* locus carrying the inducible GDV1-GFP-DD expression cassette in 3D7/iGP parasites (bottom). The relative position of the sgt_*cg6* sgRNA target sequence is shown in purple. The pD_*cg6_cam-gdv1-gfp-dd* donor plasmid contains the *gdv1-gfp-dd* fusion gene controlled by the *P. falciparum cam* promoter and *pbdhfr-ts* terminator (PbDT 3') elements, flanked on either side by a homology region (HR) for homology-directed repair (orange). The pHF_gC-*cg6* plasmid contains expression cassettes for SpCas9 (dark grey), the sgRNA (purple) and the *hdhfr-fcu* positive-negative drug selection marker (brown-grey). Primer binding sites used to confirm successful gene editing by PCR are indicated by red arrowheads. **b** Diagnostic PCRs on gDNA from NF54 wt parasites and the 3D7/iGP mother line. Primer combinations to detect the presence and absence of the *cg6* wt locus in NF54 wt and 3D7/iGP, respectively, and integration of the full *gdv1-gfp-dd* expression cassette in 3D7/iGP (left panel). Primer combinations to detect the 5' and 3' recombination events in 3D7/iGP (middle panel). Primer combinations to detect the presence of the pHF_gC-*cg6* and the pD_*cg6_cam-gdv1-gfp-dd* plasmids in 3D7/iGP (right panel). pDNA, plasmid DNA control. Results are representative of two independent experiments. **c** Diagnostic PCRs on gDNA from NF54 wt parasites, the 5-FC-treated 3D7/iGP mother line and three clones. Primer combinations to detect the presence and absence of the *cg6* wt locus in NF54 wt and 3D7/iGP parasites, respectively, and integration of the full *gdv1-gfp-dd* expression cassette in 3D7/iGP parasites (left panel). Primer combinations to detect the presence of the pD_*cg6_cam-gdv1-gfp-dd* (middle panel) and pHF_gC-*cg6* (right panel) plasmids in 3D7/iGP parasites. pDNA, plasmid DNA control. Results are representative of two independent experiments. **d** Schematic map of a pD_*cg6_cam-gdv1-gfp-dd* donor plasmid concatamer integrated into the *cg6* locus by double-crossover recombination (for reasons of simplicity, the integration of a tandem *gdv1-gfp-dd* expression cassette is shown; the actual number of integrated plasmid copies (*n*) is unknown). The *E. coli* plasmid backbone is indicated by a dashed arrow. The binding sites of the seq1 and p2 primers used to detect this event by PCR are indicated by red arrowheads. Diagnostic PCRs on gDNA from NF54 wt parasites, the 5-FC-treated 3D7/iGP mother line and three clones show the presence of an integrated pD_*cg6_cam-gdv1-gfp-dd* donor plasmid concatamer in the 5-FC-treated 3D7/iGP mother line and clone D9 but not in clones B9 and F10. Results are representative of two independent experiments.

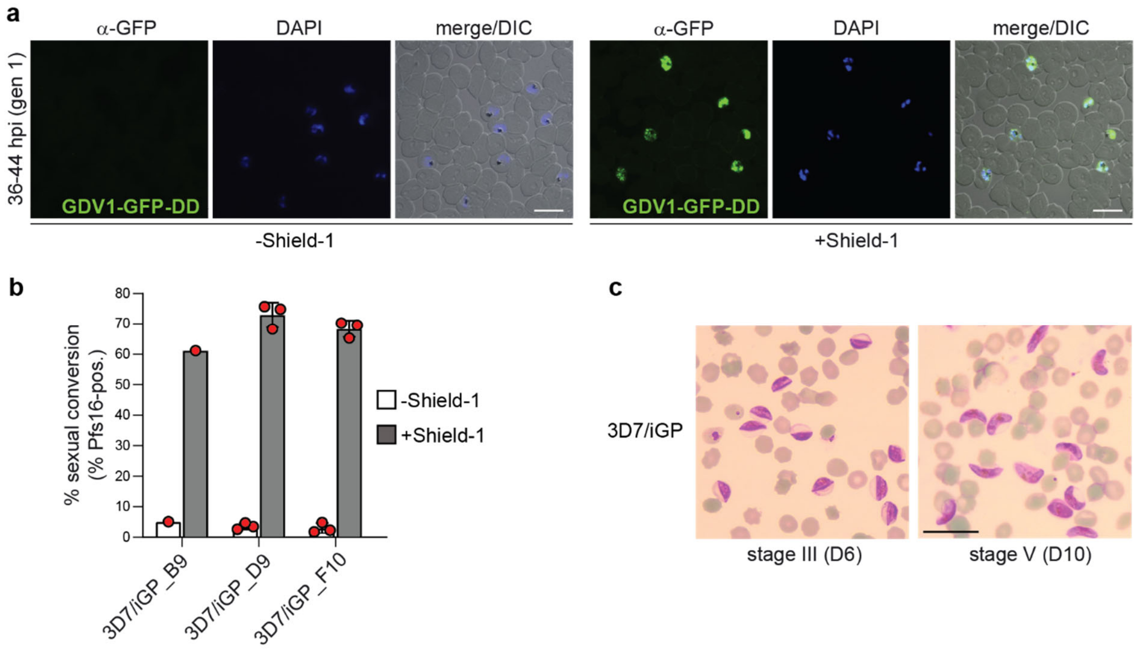

Supplementary Figure 2. Characterisation of the 3D7/iGP mother line and three 3D7/iGP clones. **a** α-GFP IFA images illustrating the Shield-1-dependent induction of GDV1-GFP-DD expression in schizonts of the 3D7/iGP mother line (36-44 hpi). Nuclei were stained with DAPI. DIC, differential interference contrast. Images are representative of three independent experiments. Scale bar, 10 µm. **b** Proportion of Pfs16-positive iRBCs (SCRs) in the progeny of untreated (−Shield-1) or Shield-1-treated 3D7/iGP clones (+Shield-1) (mean ± SD, *n* = three biologically independent experiments; one experiment for clone B9). Closed circles represent data points for individual experiments (>260 DAPI-positive cells counted per experiment). **c** Images of Giemsa-stained gametocyte cultures obtained after inducing sexual commitment in the 3D7/iGP mother line, acquired on day 6 (D6, stage III gametocytes) and day 10 (D10, stage V gametocytes) of gametocytogenesis. Images are representative of three independent experiments. Scale bar, 20 µm.

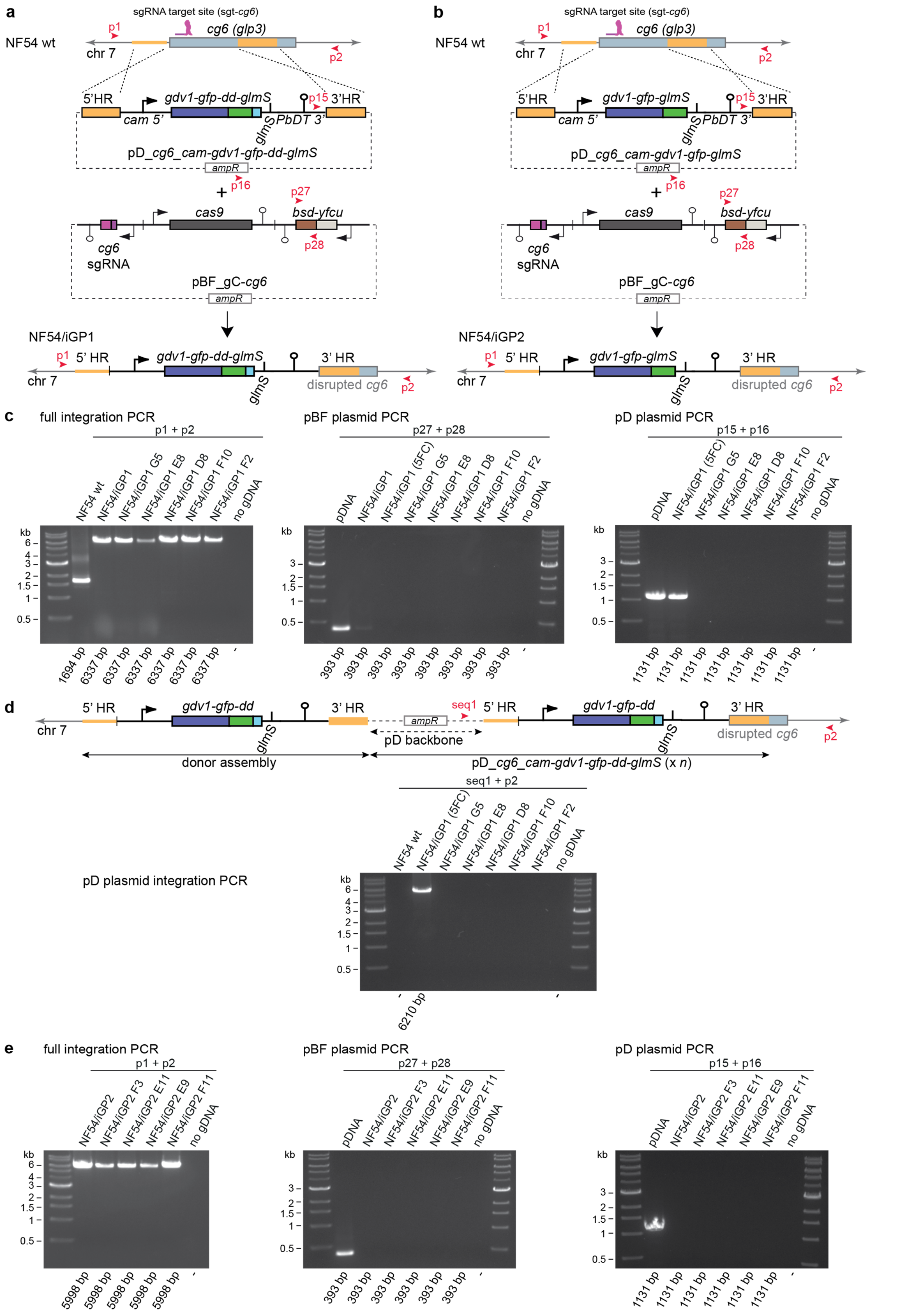

Supplementary Figure 3. CRISPR/Cas9-based engineering of the NF54/iGP lines. a Schematic maps of the endogenous *cg6* (*glp3*) locus (PF3D7_0709200) in NF54 wild type (wt) parasites (top), the

pD_*cg6_cam-gdv1-gfp-dd-glmS* donor and pBF_gC-*cg6* CRISPR/Cas9 transfection plasmids (center), and the disrupted *cg6* locus carrying the inducible GDV1-GFP-DD-glmS expression cassette in NF54/iGP1 parasites (bottom). The relative position of the sgt_*cg6* sgRNA target sequence is shown in purple. The pD_*cg6_cam-gdv1-gfp-dd-glmS* donor plasmid contains the *gdv1-gfp-dd-glmS* fusion gene controlled by the *P. falciparum cam* promoter and *P. berghei dhfr-ts* terminator (PbDT 3'), flanked on either side by a homology region (HR) for homology-directed repair (orange). The pBF_gC-*cg6* plasmid contains expression cassettes for SpCas9 (dark grey), the sgRNA (purple) and the *bsd-fcu* positive-negative drug selection marker (brown-grey). Primer binding sites used to confirm successful gene editing by PCR are indicated by red arrowheads. **b** Schematic maps of the endogenous *cg6* (*glp3*) locus (PF3D7_0709200) in NF54 wt parasites (top), the pD_*cg6_cam-gdv1-gfp-glmS* donor and pBF_gC-*cg6* CRISPR/Cas9 transfection plasmids (center), and the disrupted *cg6* locus carrying the inducible GDV1-GFP-glmS expression cassette in NF54/iGP2 parasites (bottom). **c** Diagnostic PCRs on gDNA from NF54 wt parasites, the NF54/iGP1 mother line before and after 5-FC treatment and five NF54/iGP1 clones. Primer combinations to detect the presence and absence of the *cg6* wt locus in NF54 wt and NF54/iGP1 parasites, respectively, and integration of the full *gdv1-gfp-dd* expression cassette in NF54/iGP1 parasites (left panel). Primer combinations to detect the presence of the pBF_gC-*cg6* (middle panel) and pD_*cg6_cam-gdv1-gfp-dd-glmS* (right panel) plasmids. Results are representative of two independent experiments. **d** Schematic map of a pD_*cg6_cam-gdv1-gfp-dd-glmS* donor plasmid concatamer integrated into the *cg6* locus by double-crossover recombination (for reasons of simplicity, the integration of a tandem *gdv1-gfp-dd* expression cassette is shown; the actual number of integrated plasmid copies (*n*) is unknown). The *E. coli* plasmid backbone is indicated by a dashed arrow. The binding sites of the seq1 and p2 primers used to detect this event by PCR are indicated by red arrowheads. Diagnostic PCRs on gDNA from NF54 wt parasites, the 5-FC-treated NF54//iGP1 mother line and five clones show the presence of an integrated pD_*cg6_cam-gdv1-gfp-dd-glmS* donor plasmid concatamer in the 5-FC-treated NF54/iGP1 mother line but not in any of the clones. Results are representative of two independent experiments. **e** Diagnostic PCRs on gDNA from the NF54/iGP2 mother line and four NF54/iGP2 clones. Primer combinations are as described above for panel c. Results are representative of two independent experiments.

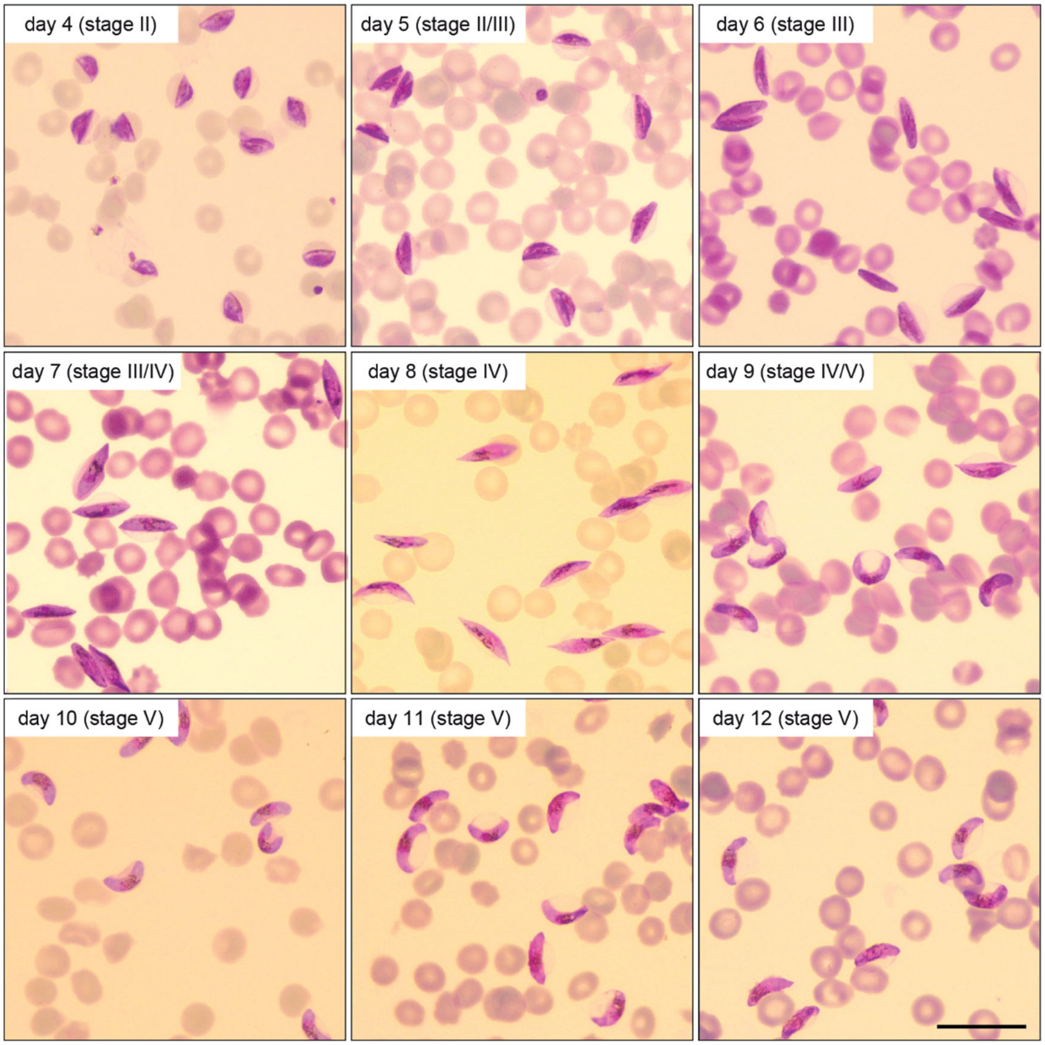

Supplementary Figure 4. Synchronous maturation of induced NF54/iGP2 gametocytes. Images of synchronously developing NF54/iGP2 gametocytes obtained after inducing sexual commitment in the previous cell cycle through removal of GlcN from the culture medium. Giemsa-stained blood smears were prepared daily from day 4 (stage II) to day 12 (stage V) of gametocytogenesis. Gametocyte cultures were treated with 50 mM GlcNAc from day 1 to 6 to eliminate asexual blood stage parasites. Images are representative of three independent experiments. Scale bar, 20 µm.

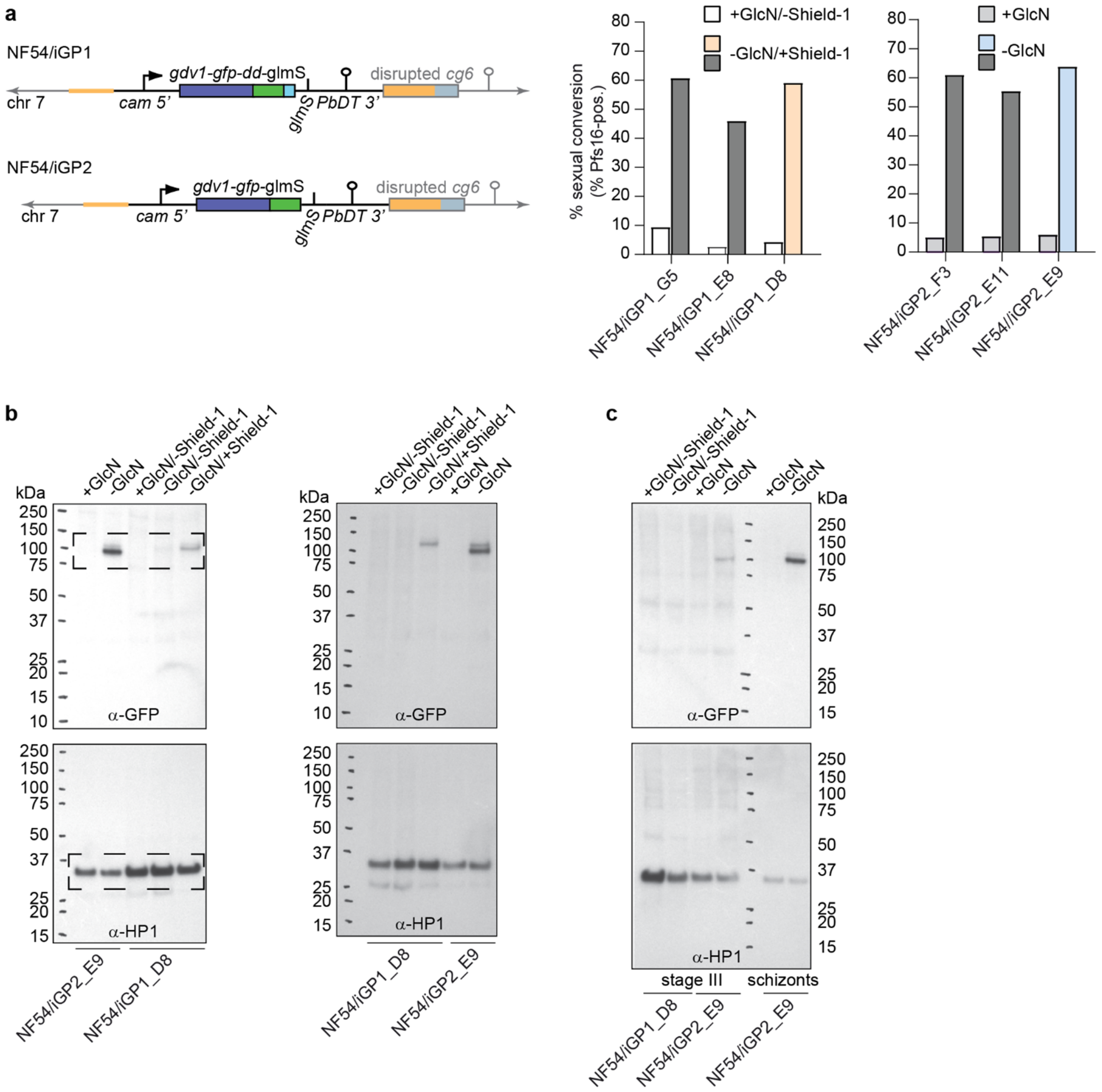

Supplementary Figure 5. SCRs in NF54/iGP1 and NF54/iGP2 clones and ectopic expression of GDV1. a Schematic map of the disrupted *cg6* (*glp3*) locus (PF3D7_0709200) carrying a single inducible GDV1-GFP-DD-glmS or GDV1-GFP-glmS expression cassette in the NF54/iGP1 or NF54/iGP2 parasite line (bottom), respectively. The 5' and 3' homology regions used for CRISPR/Cas9-based transgene insertion are positioned in the *cg6* upstream and coding sequence, respectively (orange). The proportions of Pfs16-positive iRBCs (SCRs) in the progeny of three NF54/iGP1 clones cultured under control (+GlcN/–Shield-1) and inducing conditions (–GlcN/+Shield-1) and of three NF54/iGP2 clones cultured under control (+GlcN) and inducing conditions (–GlcN) are shown on the right. Values are the result of a single experiment (≥285 DAPI-positive cells counted per experiment). Clones NF54/iGP1_D8 (orange) and NF54/iGP_E9 (blue) have been selected for further characterisation. **b** Full size Western blots showing expression of GDV1-GFP (MW=99.1 kDa) and GDV1-GFP-DD (MW=111.3 kDa) in NF54/iGP2_E9 and NF54/iGP1_D8 schizonts (34-42 hpi), respectively. Parasites were split (NF54/iGP1_D8: +GlcN/–Shield-1, –GlcN/–Shield-1 and –GlcN/+Shield-1; NF54/iGP2_E9:

+GlcN and −GlcN) 34 hours before sample collection. PfHP1 (MW=31 kDa) expression levels served as a control to compare the relative numbers of nuclei loaded per lane. Dashed boxes show the sections presented in Fig. 2f. The blots on the right show the results from a biologically independent experiment. **c** Full size Western blot showing expression of GDV1-GFP-DD (MW=111.3 kDa) and GDV1-GFP (MW=99.1 kDa) in NF54/iGP1_D8 and NF54/iGP2_E9 stage III gametocytes (day 6), respectively. NF54/iGP1_D8 and NF54/iGP2_E9 gametocytes were cultured separately under +GlcN/−Shield-1 or −GlcN/−Shield-1 conditions and under +GlcN or −GlcN conditions, respectively. Extracts from NF54/iGP2_E9 schizonts cultured under control (+GlcN) and GDV1-GFP-inducing conditions (−GlcN) were loaded as a reference. PfHP1 (MW=31 kDa) served as a control to compare the relative numbers of nuclei loaded per lane. Results are representative of a single experiment.

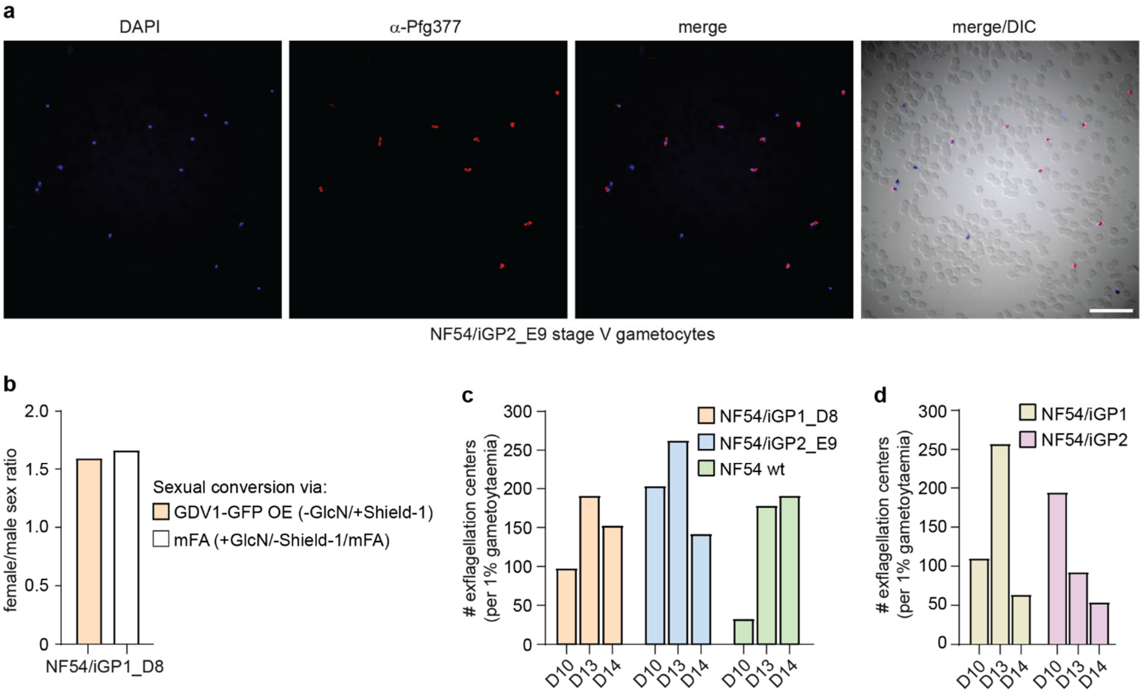

Supplementary Figure 6. Sex ratios and exflagellation of NF54/iGP1 and NF54/iGP2 gametocytes. **a** α-Pfg377 IFA images distinguishing female (DAPI-positive/Pfg377-positive) from male (DAPI-positive/Pfg377-negative) NF54/iGP2_E9 stage V gametocytes. Nuclei were stained with DAPI. DIC, differential interference contrast. Images are representative of three independent experiments. Scale bar, 50 μm. **b** Female/male sex ratios of NF54/iGP1_D8 stage V gametocytes obtained via GDV1-GFP-DD overexpression (–GlcN/+Shield1) or via induction of sexual commitment using mFA medium (+GlcN/-Shield-1/mFA) as quantified from α-Pfg377 IFAs. Values represent the results from a single experiment (≥169 gametocytes scored). **c** Exflagellation rates of NF54/iGP1_D8, NF54/iGP2_E9 and NF54 wt control stage V gametocytes determined on days 10 (D10), 13 (D13) and 14 (D14) of gametocytogenesis. Values represent the results from a single experiment. **d** Exflagellation rates of NF54/iGP1 and NF54/iGP2 stage V gametocytes determined on days 10 (D10), 13 (D13) and 14 (D14) of gametocytogenesis. Values represent the results from a single experiment.

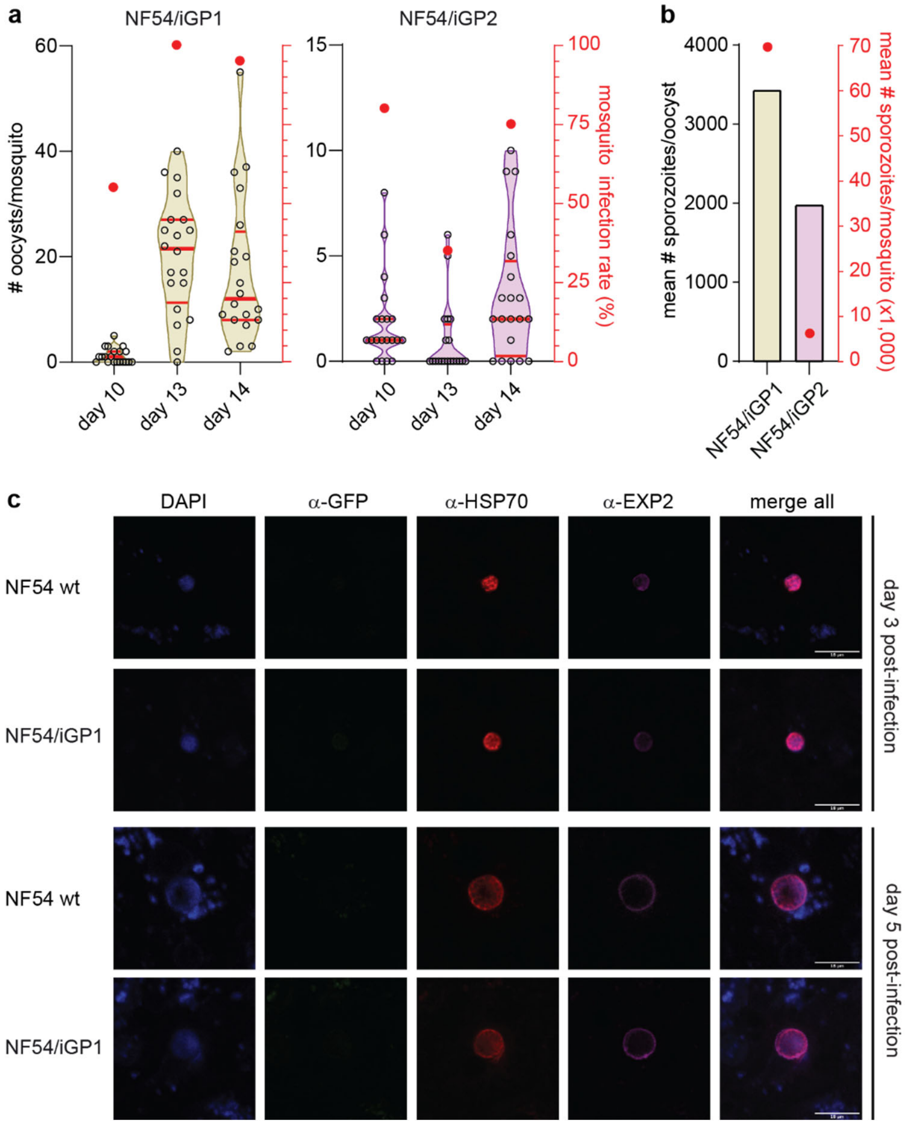

Supplementary Figure 7. Results from SMFAs and hepatocyte infection assays performed with the NF54/iGP1 and NF54/iGP2 mother lines. a NF54/iGP1 (beige) and NF54/iGP2 (purple) stage V gametocytes were fed to female *Anopheles stephensi* mosquitoes on day 10, 13 and 14 of gametocytogenesis (D10, D13, D14) in a single SMFA experiment. The Violin plots show the distribution of the number of oocysts (open circles) detected in each of the 20 mosquitoes dissected per feed (left x-axis). The median (thick red line) and upper and lower quartiles (thin red lines) are indicated. Closed red circles represent the mean oocyst prevalence (number of infected mosquitoes) determined for each feed (right y-axis). **b** Mean number of salivary gland sporozoites per oocyst (left x-axis) and per mosquito (closed red circles; right x-axis) 17 days after infection with NF54/iGP1 (beige) and NF54/iGP2 (purple) day 14 gametocytes. Values represent the results from a single experiment (≥34 mosquitoes dissected per infected batch). **c** Representative confocal microscopy IFA images showing intracellular parasites after infection of primary human hepatocytes with NF54/iGP1 and NF54 wt control sporozoites. Parasites were stained with rabbit α-PfHSP70 (cytosol; red) and mouse α-PfEXP2 antibodies (purple; parasitophorous vacuolar membrane). Nuclei were stained with DAPI. α-GFP

antibodies were used to test for potential ectopic expression of GDV1 in liver stages. Images are representative of a single experiment. Scale bar, 18 µm.

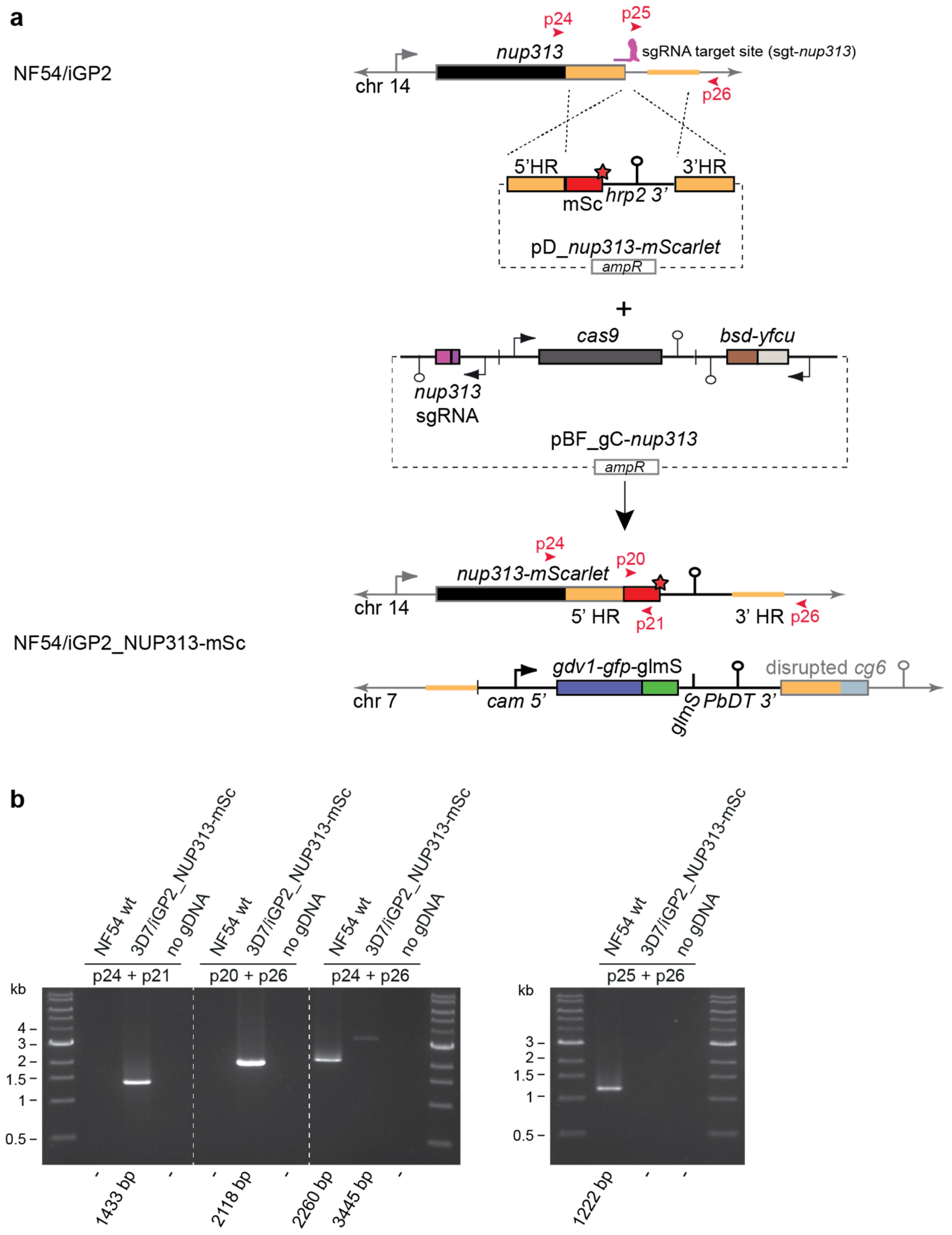

Supplementary Figure 8. CRISPR/Cas9-based engineering of the NF54/iGP2_NUP313-mSc line. **a** Schematic maps of the endogenous *nup313* locus (PF3D7_1446500) in NF54/iGP2 parasites (top), the pD_*nup313-mScarlet* donor and pBF_gC-*nup313* CRISPR/Cas9 transfection plasmids (center), and the edited *nup313* locus in NF54/iGP2_NUP313-mSc parasites expressing the NUP313-mScarlet fusion protein (bottom). The relative position of the sgt_*cg6* sgRNA target sequence is shown in purple. The pD_*nup313-mScarlet* donor plasmid contains the *mScarlet* sequence followed by the *P. falciparum* histidine-rich protein 2 (*hrp2*) terminator, flanked on either side by a homology region (HR) for homology-directed repair (orange). The 5' HR corresponds to the 3' end of *nup313* (omitting the stop codon), fused in frame to the *mScarlet* sequence. The pBF_gC-*nup313* plasmid contains expression cassettes for SpCas9 (dark grey), the sgRNA (purple) and the *bsd-fcu* positive-negative drug selection marker (brown-grey). Primer binding sites used to confirm successful gene editing by PCR are indicated

by red arrowheads. The red asterisk denotes the STOP codon. **b** Diagnostic PCRs on gDNA from NF54 wild type (wt) and NF54/iGP2_NUP313-mSc parasites. Primer combinations to detect the 5' and 3' recombination events in NF54/iGP2_NUP313-mSc and the presence and absence of the *nup313* wt locus in NF54 wt and NF54/iGP2_NUP313-mSc, respectively, and insertion of the *mScarlet-hrp2 3'* sequence in NF54/iGP2_NUP313-mSc (left panel). Alternative primer combination to detect the presence and absence of the *nup313* wt locus in NF54 wt and NF54/iGP2_NUP313-mSc, respectively (right panel). Results are representative of a single experiment.

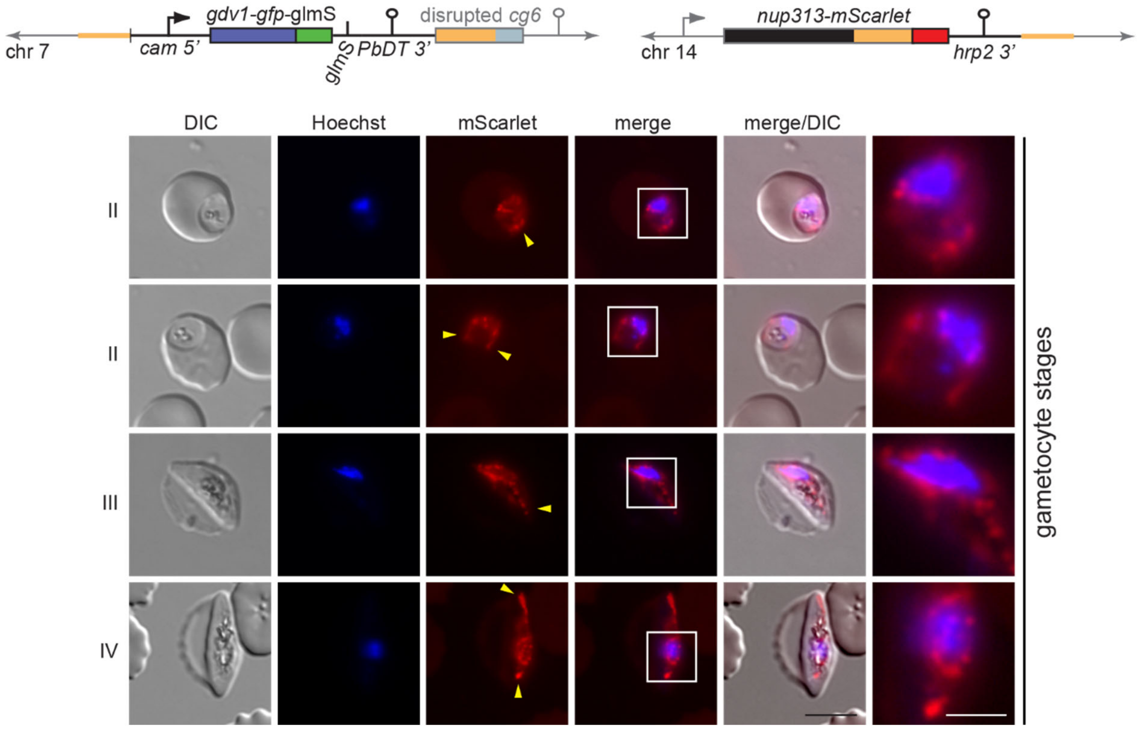

Supplementary Figure 9. Nuclei in stage II to IV gametocytes undergo marked morphological transformations. Schematic maps of the disrupted *cg6* (*glp3*) locus (PF3D7_0709200) carrying a single inducible GDV1-GFP-glmS expression cassette and the tagged *nup313* locus in the double-transgenic NF54/iGP2_NUP313-mSc line are shown on top. The 5' and 3' homology regions used for CRISPR/Cas9-based genome editing are shown in orange. Live cell fluorescence microscopy images showing the localization of NUP313-mScarlet (red) in stage II to IV gametocytes. Lateral extensions of the nucleus away from Hoechst-stained bulk chromatin are highlighted by yellow arrowheads. II-IV, stage II to IV gametocytes. DIC, differential interference contrast. Nuclei were stained with Hoechst (blue). Images are representative of four biologically independent experiments. Scale bar, 5 µm. White frames refer to the magnified view presented in the rightmost images (scale bar, 2 µm).

```
>mScarlet
atggtgagcaagggcgaggcagtgatcaaggagttcatgcggttcaaggtgcacatggagggctccatgaacggccacgagttcgagatcgagg
gcgagggcgagggccgcccctacgagggcacccagaccgccaagctgaaggtgaccaagggtggccccctgcccttctcctgggacatcctgtc
ccctcagttcatgtacggctccagggccttcaccaagcaccccgccgacatccccgactactataagcagtccttccccgagggcttcaagtgg
gagcgcgtgatgaacttcgaggacggcggcgccgtgaccgtgacccaggacacctccctggaggacggcaccctgatctacaaggtgaagctcc
gcggcaccaacttccctcctgacggccccgtaatgcagaagaagacaatgggctgggaagcgtccaccgagcggttgtaccccgaggacggcgt
gctgaagggcgacattaagatggccctgcgcctgaaggacggcggccgctacctggcggacttcaagaccacctacaaggccaagaagcccgtg
cagatgcccggcgcctacaacgtcgaccgcaagttggacatcacctcccacaacgaggactacaccgtggtggaacagtacgaacgctccgagg
gccgccactccaccggcggcatggacgagctgtacaag

>mScarlet co
agtaaaggtgaagcagttataaaagaatttatgagatttaaagtacatatggaaggttcaatgaatggacatgaatttgaaatagaaggagaag
gtgaaggaagaccatatgaaggaacacaaacagctaaattgaaagttacaaaaggtggaccattaccatttagttgggatattttatcaccaca
atttatgtatggtagtagagcatttacaaaacatccagctgatataccagattattataaacaatcatttccagaaggatttaaatgggaaaga
gtaatgaattttgaagatggaggtgcagttacagtaacacaagatacaagtttagaagatggtacattaatttataaagttaaattgagaggta
caaattttccaccagatggaccagtaatgcaaaagaaaacaatgggatgggaagcatcaacagaaagattatatccagaagatggtgttttaaa
aggagatataaaaatggctttaagattaaaagatggaggtagatatttagcagattttaaaacaacatataaagctaaaaaaccagttcaaatg
ccaggtgcttataatgtagatagaaaattggatataacaagtcataatgaagattatacagttgtagaacaatatgaaagaagtgaaggaagac
attcaacaggtggaatggatgaattatataaa


>Pairwise sequence alignment
mScarlet        atggtgagcaagggcgaggcagtgatcaaggagttcatgcggttcaaggtgcacatggag 60
mScarlet co     ------agtaaaggtgaagcagttataaaagaatttatgagatttaaagtacatatggaa 54
                      ** ** ** ** ***** ** ** ** ** *** * ** ** ** ** *****
mScarlet        ggctccatgaacggccacgagttcgagatcgagggcgagggcgagggccgcccctacgag 120
mScarlet co     ggttcaatgaatggacatgaatttgaaatagaaggagaaggtgaaggaagaccatatgaa 114
                ** ** ***** ** ** ** ** ** ** ** ** ** ** ** **  * ** ** **
mScarlet        ggcacccagaccgccaagctgaaggtgaccaagggtggccccctgcccttctcctgggac 180
mScarlet co     ggaacacaaacagctaaattgaaagttacaaaaggtggaccattaccatttagttgggat 174
                ** ** ** ** ** **  **** ** ** ** ***** **  * ** **    *****
mScarlet        atcctgtcccctcagttcatgtacggctccagggccttcaccaagcaccccgccgacatc 240
mScarlet co     attttatcaccacaatttatgtatggtagtagagcatttacaaaacatccagctgatata 234
                **  * ** ** ** ** ***** **    ** ** ** ** ** ** ** ** ** **
mScarlet        cccgactactataagcagtccttccccgagggcttcaagtgggagcgcgtgatgaacttc 300
mScarlet co     ccagattattataaacaatcatttccagaaggatttaaatgggaaagagtaatgaatttt 294
                ** ** ** ***** ** ** ** ** ** ** ** ** *****  * ** ***** **
mScarlet        gaggacggcggcgccgtgaccgtgacccaggacacctccctggaggacggcaccctgatc 360
mScarlet co     gaagatggaggtgcagttacagtaacacaagatacaagtttagaagatggtacattaatt 354
                ** ** ** ** ** ** ** ** ** ** ** **     * ** ** ** **  * **
mScarlet        tacaaggtgaagctccgcggcaccaacttccctcctgacggccccgtaatgcagaagaag 420
mScarlet co     tataaagttaaattgagaggtacaaattttccaccagatggaccagtaatgcaaaagaaa 414
                ** ** ** **  *  * ** ** ** ** ** ** ** ** ** ******** *****
mScarlet        acaatgggctgggaagcgtccaccgagcggttgtaccccgaggacggcgtgctgaagggc 480
mScarlet co     acaatgggatgggaagcatcaacagaaagattatatccagaagatggtgttttaaaagga 474
                ******** ******** ** ** **  * ** ** ** ** ** ** **  * ** **
mScarlet        gacattaagatggccctgcgcctgaaggacggcggccgctacctggcggacttcaagacc 540
mScarlet co     gatataaaaatggctttaagattaaaagatggaggtagatatttagcagattttaaaaca 534
                ** ** ** *****  *   *   * ** ** ** **  * **  * ** ** ** ** **
mScarlet        acctacaaggccaagaagcccgtgcagatgcccggcgcctacaacgtcgaccgcaagttg 600
mScarlet co     acatataaagctaaaaaaccagttcaaatgccaggtgcttataatgtagatagaaaattg 594
                ** ** ** ** ** ** ** ** ** ***** ** ** ** ** ** **  * ** ***
mScarlet        gacatcacctcccacaacgaggactacaccgtggtggaacagtacgaacgctccgagggc 660
mScarlet co     gatataacaagtcataatgaagattatacagttgtagaacaatatgaaagaagtgaagga 654
                ** ** **    ** ** ** ** ** ** ** ** ***** ** *** *    ** **
mScarlet        cgccactccaccggcggcatggacgagctgtacaag                         696
mScarlet co     agacattcaacaggtggaatggatgaattatataaa                         690
                 * ** ** ** ** ***** **  * ** **
```

Supplementary Figure 10. Pairwise alignment of the *mScarlet* and *P. falciparum* codon-optimised *mScarlet* gene sequences. Nucleotide sequences and Clustal Omega (https://www.ebi.ac.uk/Tools/msa/clustalo/) output of the pairwise sequence alignment of *mScarlet* and the *P. falciparum* codon-optimised version of *mScarlet* (mScarlet co). Asterisks denote identical nucleotides.

Supplementary Table 1. Oligonucleotides used in this study.

Application	Primer name	target sequence	Sequence (5'-3')
PCRs to clone transfection vectors	1F	*cam* promoter	gaataaataaatataaatatgaacatgatcttataaggaaattccc
	1R	*dd* cds	gaacattaagctgccatatcctcattccagttttagaagc
	2F	*pbdhfr-ts* terminator	gagcttctaaaactggaatgaggatatggcagcttaatg
	2R	*pbdhfr-ts* terminator	tcatgctaccctgaagaagaaaag
	3F	*cg6* cds	cttcttcagggtagcatgaacatgtaaaagataaaaatg
	3R	*cg6* cds	cctcttcgctattacgccagcaacttgtctatgccacc
	4F	plasmid backbone	ctggcgtaatagcgaagagg
	4R	plasmid backbone	cattaatgaatcggccaacg
	5F	*cg6* upstream	gggaatttccttataagatcatgttcatatttatatttatttattcatttgtttg
	5R	*cg6* upstream	cgttggccgattcattaatgcacatattcgctccttc
	7F	*gfp/dd cds*	ggatgaactatacaaaaccggttctagtatgggagtgcaggtggaaac
	7R	*glmS*	cgaacattaagctgccatatccgctagcatttttcttcctcc
	8F	*pbdhfr-ts* terminator	aggaggaagaaaaatgctagcggatatggcagcttaatgttcg
	8R	*cg6* cds	ctcttctactctttcgaattcaccatgttcatcttttatacatttatc
	9F	*glmS/pbdhfr-ts* terminator	aggaggaagaaaaatgctagcggatatggcagcttaatgttcg
	9R	*gfp* cds	ctattgagaaaataagaacaagattatttgtatagttcatccatgccatg
	10F	*glmS* sequence	gcatggatgaactatacaaataatcttgttcttattttctcaatag
	10R	*glmS* sequence	cgaacattaagctgccatatccgctagcatttttcttcctcctaagattg
	13F	*nup313* cds	agtgagcgaggaagcggaagctgtttctaattcaagatctgattcc
	13R	*nup313* cds	acttgtggatccaccactactatttatcatattttgattcataaatttatgcc
	14F	*mScarlet* cds	atagtagtggtggatccacaagtaaaggagaagcagttataaaag
	14R	*mScarlet* cds	tctattattaaataaatttatttgtatagttcatccattccacc
	15F	*pfhrpII* terminator	ggaatggatgaactatacaaataaatttatttaataatagattaaaaatattataaaaataaaaac
	15R	*pfhrpII* terminator	gcactttgtagagataagtaatttaataaatatgttcttatatataatgag
	16F	*nup313* downstream	gaacatatttattaaattacttatctctacaaagtgcacacac
	16R	*nup313* downstream	gcaccatatgcgaaggagaaatttcctttgaggac
	17F	plasmid backbone	gtcctcaaaggaaatttctccttcgcatatggtgcactctcagtac
	17R	plasmid backbone	gaatcagatcttgaattagaaacagcttccgcttcctcgctcac
sgRNA annealing	11F	sgt_*cg6*	tattgcacaaatataaattaaatt
	11R	sgt_*cg6*	aaacaatttaatttatatttgtgc
	18F	*nup313 downstream*	aaactacttatctctacaaagtgc
	18R	*nup313 downstream*	tattgcactttgtagagataagta
PCRs to verify gene editing	p1	*cg6* upstream	atgtagcccatgaaagagttatg
	p2	*cg6* downstream	cacaagcacataatggtggg
	p3	*cam* promoter (only in pD)	gcatgcaagcttcgatcc
	p4	*pbdhfr-ts* terminator	gctcaattctttatgtccacaac
	p5	*cg6* cds	ggtagagttcaattcatcaaacc
	p6	*cg6* cds	gatcctgggtaacttcacag
	p10	pHF/pBF/pD backbone	gtactgagagtgcaccatatgc
	p15	*pbdhfr-ts* terminator	gatattgagcagaggatatgc
	p16	pHF/pBF/pD backbone	gcacccaactgatcttcagc
	p17	*cam* promoter (only in pHF/pBF, not in pD)	gctcgcaaatggccaaataag
	p19	*hdhfr* cds	ccttgtggaggttccttgag
	p20	*mScarlet* cds	ggaggtgcagttacagtaacacaag
	p21	*mScarlet* cds	gcattactggtccatctggtgga
	p24	*nup313* cds	tgagcatatagtaccatcagaatgg
	p25	*nup313* downstream	acacaataaaatgttcacggaataatg
	p26	*nup313* downstream (Pf3D7_1446600 cds)	gatacaagggaaggaatacaacg
	p27	*bsd* cds	atggcacctttgtctcaagaag
	p28	*bsd* cds	accctcccacacataaccag
	seq 1	pD backbone (except pD_*nup313-mScarlet*)	gcgaggaagcggaagagc

Supplementary Table 2. Summary of NF54/iGP1_D8 and NF54/iGP2_E9 properties and potential applications for future research.

**Properties**	**NF54/iGP1_D8**	**NF54/iGP2_E9**
Marker-free	yes	yes
Plasmid-free	yes	yes
Conditional ectopic GDV1 expression system	GDV1-GFP-DD-glmS	GDV1-GFP-glmS
Propagation of feeder cultures	-Shield-1/+GlcN	+GlcN
Background sexual commitment rates in feeder cultures	8%	9%
Induction of ectopic GDV1 expression	+Shield-1/-GlcN	-GlcN [a)]
Sexual commitment rates upon induction of ectopic GDV1 expression	63%	73% [b)]
Female/male sex ratio	~1.5	~2
Mosquito infection rates (%) (day 10/13/14 feed)	97.5/97.5/87.5	92.5/100/100 [c)]
Median oocysts/mosquito (day 10/13/14 feed)	6/13/8	8/37/47 [c)]
Mean salivary gland sporozoites/mosquito	70,000	100,000 [c)]
Mean salivary gland sporozoite/oocyst (day 14 feed)	4,000	1,900
Sporozoites infectious to hepatocytes	yes	yes
Ectopic GDV1 expression in gametocytes (-Shield-1/-GlcN conditions)	absent [d)]	weak; prevented by +GlcN
Ectopic GDV1 expression in mosquito and liver stages	absent	absent
**Recommendations for research [e)]**	**NF54/iGP1_D8**	**NF54/iGP2_E9**
Further genetic engineering	+++	+++
Sexual commitment	++	+++
Gametocyte biology	+++	+++
Gametocyte -omics	+++	+++
High-throughput gametocytocidal drug screening	+++	+++
Pre-clinical transmission-blocking drugs/vaccines	+++	+++
Mosquito stage biology	++	+++
Mosquito stage -omics	++	+++
Liver stage biology	++	+++
in vitro production of mosquito stage parasites	++	+++
Gametocyte biology *in vivo* (humanised mouse models) [f)]	+++	-

Footnotes: [a)] No Shield-1 needed for work with NF54/iGP2_E9 parasites. [b)] Higher sexual conversion rates achieved with NF54/iGP2_E9 parasites. [c)] Superior mosquito infection outcomes with NF54/iGP2_E9 gametocytes. [d)] Addition of GlcN not required to prevent low level expression of ectopic GDV1 in NF54/iGP1_D8 gametocytes. [e)] Recommendations reflect the authors' personal estimation and expectations about the suitability and potential applications of NF54/iGP1_D8 and NF54/iGP2_E9 parasites to support future basic and applied research, based on the results obtained during this study. [f)] As the DD/Shield-1 system is amenable to conditional protein expression *in vivo*, NF54/iGP1_D8 parasites may be suitable for mass production of gametocytes in *P. falciparum* mouse models.

Supplementary References

1 Walker, M. P. & Lindner, S. E. Ribozyme-mediated, multiplex CRISPR gene editing and CRISPR interference (CRISPRi) in rodent-infectious Plasmodium yoelii. **294**, 9555-9566 (2019).

2 Mogollon, C. M. *et al.* Rapid Generation of Marker-Free P. falciparum Fluorescent Reporter Lines Using Modified CRISPR/Cas9 Constructs and Selection Protocol. *PLoS. ONE* **11**, e0168362 (2016).

3 Bruce, M. C., Carter, R. N., Nakamura, K., Aikawa, M. & Carter, R. Cellular location and temporal expression of the Plasmodium falciparum sexual stage antigen Pfs16. *Mol. Biochem. Parasitol* **65**, 11-22 (1994).

4 Ponnudurai, T., Lensen, A. H., Meis, J. F. & Meuwissen, J. H. Synchronization of Plasmodium falciparum gametocytes using an automated suspension culture system. *Parasitology* **93 (Pt 2)**, 263-274 (1986).

5 Fivelman, Q. L. *et al.* Improved synchronous production of Plasmodium falciparum gametocytes in vitro. *Mol. Biochem. Parasitol* **154**, 119-123 (2007).
