# Supplementary material for: CRISPR/Cas9-engineered inducible gametocyte producer lines as a valuable tool for Plasmodium falciparum malaria transmission research
Source: Nat Commun. 2021 Aug 10;12:4806. doi: 10.1038/s41467-021-24954-4 (PMC8355313; doi:10.1038/s41467-021-24954-4)
Supplement: Supplementary file 3 — Reporting Summary [file 41467_2021_24954_MOESM3_ESM.pdf]

nature research

Corresponding author(s): Till Voss

Last updated by author(s): Jun 24, 2021

Reporting Summary

Nature Research wishes to improve the reproducibility of the work that we publish. This form provides structure for consistency and transparency in reporting. For further information on Nature Research policies, see our Editorial Policies and the Editorial Policy Checklist.

Statistics

For all statistical analyses, confirm that the following items are present in the figure legend, table legend, main text, or Methods section.

n/a	Confirmed	
☐	☒	The exact sample size (n) for each experimental group/condition, given as a discrete number and unit of measurement
☐	☒	A statement on whether measurements were taken from distinct samples or whether the same sample was measured repeatedly
☐	☒	The statistical test(s) used AND whether they are one- or two-sided<br>*Only common tests should be described solely by name; describe more complex techniques in the Methods section.*
☒	☐	A description of all covariates tested
☒	☐	A description of any assumptions or corrections, such as tests of normality and adjustment for multiple comparisons
☐	☒	A full description of the statistical parameters including central tendency (e.g. means) or other basic estimates (e.g. regression coefficient) AND variation (e.g. standard deviation) or associated estimates of uncertainty (e.g. confidence intervals)
☐	☒	For null hypothesis testing, the test statistic (e.g. F, t, r) with confidence intervals, effect sizes, degrees of freedom and P value noted<br>*Give P values as exact values whenever suitable.*
☒	☐	For Bayesian analysis, information on the choice of priors and Markov chain Monte Carlo settings
☒	☐	For hierarchical and complex designs, identification of the appropriate level for tests and full reporting of outcomes
☒	☐	Estimates of effect sizes (e.g. Cohen's d, Pearson's r), indicating how they were calculated

Our web collection on statistics for biologists contains articles on many of the points above.

Software and code

Policy information about availability of computer code

Data collection	Microscopy images obtained with the Leica DM5000 B fluorescence microscope were acquired with the Leica application suite software (LAS 4.9.0 or LAS X) and processed using Adobe Photoshop CC, ImageJ (version 1.52n) or Fiji. Microscopy images obtained with the Leica DM6 B fluorescence microscope were acquired with the Leica application suite software (LAS X) and processed using Adobe Photoshop CS2. Microscopy images obtained with the Zeiss LSM880 microscope were acquired with the Zen Black software (version 14.0.18.201) and processed using Fiji.
Data analysis	All data have been analysed using Microsoft Excel 2016 or GraphPad Prism (version 8.2.1).

For manuscripts utilizing custom algorithms or software that are central to the research but not yet described in published literature, software must be made available to editors and reviewers. We strongly encourage code deposition in a community repository (e.g. GitHub). See the Nature Research guidelines for submitting code & software for further information.

Data

Policy information about availability of data

All manuscripts must include a data availability statement. This statement should provide the following information, where applicable:

- Accession codes, unique identifiers, or web links for publicly available datasets
- A list of figures that have associated raw data
- A description of any restrictions on data availability

All data generated or analysed during this study are included in this published article and its associated Supplementary Information and Source data files. The associated Source data file contains the raw data underlying the graphs presented in Figs 1d, 2b, 2e, 2g, 3a, 3b and Supplementary Figs. 2b, 5a, 6b-d, 7a-b). Correspondence and requests for materials should be addressed to T.S.V.

Field-specific reporting

Please select the one below that is the best fit for your research. If you are not sure, read the appropriate sections before making your selection.

- [x] Life sciences
- [ ] Behavioural & social sciences
- [ ] Ecological, evolutionary & environmental sciences

For a reference copy of the document with all sections, see nature.com/documents/nr-reporting-summary-flat.pdf

Life sciences study design

All studies must disclose on these points even when the disclosure is negative.

Sample size	Sample sizes were chosen according to standards in the field. Most experiments were repeated three times independently, except for experiments conducted to confirm findings by a complementary approach or with independent cell lines, which were performed once or twice independently (see below) for details. The exact number of cells analysed in each experiment is provided in the Source data table (Figs 1d, 2b, 2e, 2g, 3a, 3b, S2b, S5a, S6b-d, S7a-b).<br>For microscopy-based quantification of sexual conversion rates or gametocyte sex ratios, over 100 parasites have been scored per condition and experiment (e.g. Filarsky et al., Science 2018, PMID: 29590075; Portugaliza et al., eLife 2020, PMID: 33084568; Bancells et al., Nat Microbiol 2019, PMID: 30478286). Western blots have been repeated twice using protein extracts from biologically independent samples (e.g. Bertschi et al., Nat Microbiol 2017). For each gametocyte sample analysed by SMFA, 20 individual mosquitoes were dissected (e.g. Lensen et al., Trans R Soc Trop Med Hyg 1996, PMID: 8730302; Stone et al., Nat Commun 2018, PMID: 29422648). Live cell fluorescence microscopy of NUP313-expressing parasites has been performed on three independent batches of parasites for each stage, and each time 50-100 cells have been viewed per sample.
Data exclusions	No data were excluded.
Replication	All experiments in this study were replicated successfully three times independently (as stated in the figure legends), except for experiments conducted to confirm findings by a complementary approach or with independent cell lines:<br>- sexual conversion rate assays performed with the 3D7/iGP upon treatment with 337.5 nM Shield-1 (two replicates; Fig. 1d).<br>- Western blots of schizont extracts (two replicates; Figs. 2f and S5b) and gametocyte extracts (one experiment; Figs. S5c).<br>- standard membrane feeding assays (SMFAs) performed with (1) the NF54/iGP clones and NF54 wt control day 14 sample (two replicates; Fig. 3a); and (2) the NF54 wt control day 13 and day 14 samples and the NF54/iGP mother lines (one experiment; Figs. 3a and S7a).<br>- quantification of salivary gland sporozoites (one experiment for each parasite line; Figs. 3b and S7b) and hepatocyte infection experiments (one experiment for each parasite line; Figs. 3c and S7c).<br>- exflagellation assays (one experiment each on three consecutive days for each parasite line; Fig. S6c).<br>- sex ratio determination of NF54/iGP1_D8 (one experiment; Fig. S6b).
Randomization	No experimental groups were involved hence randomization was not relevant to this study.
Blinding	No experimental groups were involved hence blinding was not relevant to this study.

Reporting for specific materials, systems and methods

We require information from authors about some types of materials, experimental systems and methods used in many studies. Here, indicate whether each material, system or method listed is relevant to your study. If you are not sure if a list item applies to your research, read the appropriate section before selecting a response.

Materials & experimental systems

n/a	Involved in the study	
	x	Antibodies
	x	Eukaryotic cell lines
x		Palaeontology and archaeology
x		Animals and other organisms
x		Human research participants
x		Clinical data
x		Dual use research of concern

Methods

n/a	Involved in the study	
x		ChIP-seq
x		Flow cytometry
x		MRI-based neuroimaging

Antibodies

Antibodies used	Primary antibodies:<br>- mouse mAb α-GFP from Roche Diagnostics (#11814460001) was used at a 1:200-1,000 dilution.<br>- mouse mAb α-Pfs16 (Moelans, PhD Thesis, Radboud University, ISBN 90–9007799-9007795, 1995) was used at a 1:500 dilution.<br>- rabbit α-PfHP1 (Brancucci et al., Cell Host Microbe 2014; PMID: 25121746) was used at a 1:14,000 dilution.<br>- rabbit α-PfHsp70 from StressMarq Biosciences (SPC-186) was used at a 1:75 dilution.

	- mouse mAb α-PfExp2 from The European Malaria Reagent Repository was used at a 1:1,000 dilution.<br>- chicken α-GFP from Invitrogen (#A10262) was used at a 1:1,000 dilution.<br>- rabbit α-Pfg377 (Alano et al., Mol Biochem Parasitol 1995; PMID: 8719156) was used at a 1:1,000 dilution.<br>Secondary antibodies:<br>- goat-α-mouse IgG (H&L)-Alexa Fluor 488 from Invitrogen (#A-11001) was used at a 1:200 dilution.<br>- sheep α-mouse IgG (H&L)-HRP from GE Healthcare (#NXA931) was used at a 1:10,000 dilution.<br>- donkey α-rabbit IgG (H&L)-HRP from GE Healthcare (#NA934) was used at a 1:10,000 dilution.<br>- goat α-chicken IgY-Alexa Fluor Plus 488 from Invitrogen (#A32931) was used at a 1:400 dilution.<br>- goat α-rabbit IgG (H&L)-Alexa Fluor 594 from Invitrogen (#A32740) was used at a 1:400 dilution.<br>- goat α-mouse IgG (H&L)-Alexa Fluor 647 from Invitrogen (#A32728) was used at a 1:400 dilution.<br>- goat α-rabbit IgG (H&L)-Alexa Fluor 568 from Invitrogen (#A-11011) was used at a 1:400 dilution.
Validation	- mouse mAb α-Pfs16 has been published by Moelans (Moelans IIMD. Pfs16, a potential vaccine candidate against the human malaria parasite Plasmodium falciparum. PhD thesis, Radboud University, ISBN 90–9007799-9007795, 1995; https://repository.ubn.ru.nl/handle/2066/145921) and has since been used in several other studies (e.g. PMID: 29590075, PMID: 25121746, PMID: 32577509, PMID: 30478286).<br>- rabbit α-PfHP1 has been published and validated (Brancucci et al., Cell Host Microbe 2014; PMID: 25121746).<br>- rabbit α-Pfg377 has been published and validated (Alano et al., Mol Biochem Parasitol 1995; PMID: 8719156).<br><br>Commercially available primary antibodies (validation information is available from the suppliers):<br>- mouse mAb α-GFP from Roche Diagnostics (#11814460001) (https://www.sigmaaldrich.com/CH/en/product/roche/11814460001).<br>- rabbit α-PfHsp70 from StressMarq Biosciences (SPC-186) (https://www.stressmarq.com/?post_type=product&s=spc-186&v=3e8d115eb4b3).<br>- mouse mAb α-PfExp2 from The European Malaria Reagent Repository (http://www.malariaresearch.eu/reagents/monoclonal-antibody/77-anti-exp-2).<br>- chicken α-GFP from Invitrogen (#A10262) (https://www.thermofisher.com/antibody/product/GFP-Antibody-Polyclonal/A10262).

Eukaryotic cell lines

Policy information about cell lines

Cell line source(s)	The P. falciparum wild type reference strain 3D7 (Walliker et al., Science 1987; PMID: 3299700) was obtained from Alan Cowman (The Walter and Eliza Hall Institute of Medical Research, Melbourne, Australia). The P. falciparum wild type NF54 strain (Delemarre and van der Kaay, Ned Tijdschr Geneeskd 1979; PMID: 390409) was provided by the Sauerwein lab.
Authentication	None of the cell lines were authenticated in our lab.
Mycoplasma contamination	The cell lines were not tested for mycoplasma contamination.
Commonly misidentified lines (See ICLAC register)	No commonly misidentified lines were used in this study.
